# Supplementary material for: HIVprotI: an integrated web based platform for prediction and design of HIV proteins inhibitors
Source: J Cheminform. 2018 Mar 9;10:12. doi: 10.1186/s13321-018-0266-y (PMC5845081; doi:10.1186/s13321-018-0266-y)
Supplement: Supplementary file 2 — Additional file 2. Source code of HIVProtI web server. [file 13321_2018_266_MOESM2_ESM.zip › HIVprotI_Source-code/hivprotI/weka-3-6-6/doc/weka/clusterers/CLOPE.html]

CLOPE


---


|  |  |  |
| --- | --- | --- |
| **Overview** \| **Package** \| **Class** \| **Tree** \| **Deprecated** \| **Index** \| **Help** \| **Weka's home** | |  |
| **PREV CLASS**   **NEXT CLASS** | **FRAMES**    **NO FRAMES**     **All Classes** |  |
| SUMMARY: NESTED \| FIELD \| CONSTR \| METHOD | DETAIL: FIELD \| CONSTR \| METHOD |  |


---


## weka.clusterers Class CLOPE

```
java.lang.Object
  weka.clusterers.AbstractClusterer
      weka.clusterers.CLOPE
```

**All Implemented Interfaces:**: java.io.Serializable, java.lang.Cloneable, Clusterer, CapabilitiesHandler, OptionHandler, RevisionHandler, TechnicalInformationHandler

---

``` public class CLOPE extends AbstractClusterer implements OptionHandler, TechnicalInformationHandler ```

Yiling Yang, Xudong Guan, Jinyuan You: CLOPE: a fast and effective clustering algorithm for transactional data. In: Proceedings of the eighth ACM SIGKDD international conference on Knowledge discovery and data mining, 682-687, 2002.


BibTeX:

```
 @inproceedings{Yang2002,
    author = {Yiling Yang and Xudong Guan and Jinyuan You},
    booktitle = {Proceedings of the eighth ACM SIGKDD international conference on Knowledge discovery and data mining},
    pages = {682-687},
    publisher = {ACM  New York, NY, USA},
    title = {CLOPE: a fast and effective clustering algorithm for transactional data},
    year = {2002}
 }
```


Valid options are:

```
 -R <num>
  Repulsion
  (default 2.6)
```

**Version:**
:   $Revision: 5538 $

**Author:**
:   Alexander Smirnov (austellus@gmail.com)

**See Also:**: Serialized Form

---

| **Field Summary** | |
| --- | --- |
| `java.util.ArrayList<weka.clusterers.CLOPE.CLOPECluster>` | `clusters`             Array of clusters |


**Constructor Summary**

`CLOPE()`
  
          the default constructor


| **Method Summary** | |
| --- | --- |
| `int` | `AddInstanceToBestCluster(Instance inst)`             Add instance to best cluster |
| `void` | `buildClusterer(Instances data)`             Generate Clustering via CLOPE |
| `int` | `clusterInstance(Instance instance)`             Classifies a given instance. |
| `Capabilities` | `getCapabilities()`             Returns default capabilities of the clusterer. |
| `java.lang.String[]` | `getOptions()`             Gets the current settings of CLOPE |
| `double` | `getRepulsion()`             gets the repulsion |
| `java.lang.String` | `getRevision()`             Returns the revision string. |
| `TechnicalInformation` | `getTechnicalInformation()`             Returns an instance of a TechnicalInformation object, containing detailed information about the technical background of this class, e.g., paper reference or book this class is based on. |
| `java.lang.String` | `globalInfo()`             Returns a string describing this DataMining-Algorithm |
| `java.util.Enumeration` | `listOptions()`             Returns an enumeration of all the available options.. |
| `static void` | `main(java.lang.String[] argv)`             Main method for testing this class. |
| `int` | `MoveInstanceToBestCluster(Instance inst)`             Move instance to best cluster |
| `int` | `numberOfClusters()`             Returns the number of clusters. |
| `java.lang.String` | `repulsionTipText()`             Returns the tip text for this property |
| `void` | `setOptions(java.lang.String[] options)`             Parses a given list of options. |
| `void` | `setRepulsion(double value)`             set the repulsion |
| `java.lang.String` | `toString()`             return a string describing this clusterer |

**Methods inherited from class weka.clusterers.AbstractClusterer**

`distributionForInstance, forName, makeCopies, makeCopy`

**Methods inherited from class java.lang.Object**

`equals, getClass, hashCode, notify, notifyAll, wait, wait, wait`

**Field Detail**

### clusters

```
public java.util.ArrayList<weka.clusterers.CLOPE.CLOPECluster> clusters
```

:   Array of clusters


**Constructor Detail**

### CLOPE

```
public CLOPE()
```

:   the default constructor


**Method Detail**

### numberOfClusters

```
public int numberOfClusters()
```

:   **Description copied from class: `AbstractClusterer`**
:   Returns the number of clusters.

    :   **Specified by:**: `numberOfClusters` in interface `Clusterer` **Specified by:**: `numberOfClusters` in class `AbstractClusterer`
    :   **Returns:**: the number of clusters generated for a training dataset.

---


### listOptions

```
public java.util.Enumeration listOptions()
```

:   **Description copied from interface: `OptionHandler`**
:   Returns an enumeration of all the available options..

    :   **Specified by:**: `listOptions` in interface `OptionHandler`
    :   **Returns:**: an enumeration of all available options.

---


### setOptions

```
public void setOptions(java.lang.String[] options)
                throws java.lang.Exception
```

:   Parses a given list of options.


    Valid options are:

    ```
     -R <num>
      Repulsion
      (default 2.6)
    ```

    :   **Specified by:**: `setOptions` in interface `OptionHandler`
    :   **Parameters:**: `options` - the list of options as an array of strings **Throws:**: `java.lang.Exception` - if an option is not supported

---


### getOptions

```
public java.lang.String[] getOptions()
```

:   Gets the current settings of CLOPE

    :   **Specified by:**: `getOptions` in interface `OptionHandler`
    :   **Returns:**: an array of strings suitable for passing to setOptions()

---


### repulsionTipText

```
public java.lang.String repulsionTipText()
```

:   Returns the tip text for this property

    :   **Returns:**: tip text for this property suitable for displaying in the explorer/experimenter gui

---


### setRepulsion

```
public void setRepulsion(double value)
```

:   set the repulsion

    :   **Parameters:**: `value` - the repulsion **Throws:**: `java.lang.Exception` - if number of clusters is negative

---


### getRepulsion

```
public double getRepulsion()
```

:   gets the repulsion

    :   **Returns:**: the repulsion

---


### getCapabilities

```
public Capabilities getCapabilities()
```

:   Returns default capabilities of the clusterer.

    :   **Specified by:**: `getCapabilities` in interface `Clusterer` **Specified by:**: `getCapabilities` in interface `CapabilitiesHandler` **Overrides:**: `getCapabilities` in class `AbstractClusterer`
    :   **Returns:**: the capabilities of this clusterer **See Also:**: `Capabilities`

---


### buildClusterer

```
public void buildClusterer(Instances data)
                    throws java.lang.Exception
```

:   Generate Clustering via CLOPE

    :   **Specified by:**: `buildClusterer` in interface `Clusterer` **Specified by:**: `buildClusterer` in class `AbstractClusterer`
    :   **Parameters:**: `instances` - The instances that need to be clustered **Throws:**: `java.lang.Exception` - If clustering was not successful

---


### AddInstanceToBestCluster

```
public int AddInstanceToBestCluster(Instance inst)
```

:   Add instance to best cluster

---


### MoveInstanceToBestCluster

```
public int MoveInstanceToBestCluster(Instance inst)
```

:   Move instance to best cluster

---


### clusterInstance

```
public int clusterInstance(Instance instance)
                    throws java.lang.Exception
```

:   Classifies a given instance.

    :   **Specified by:**: `clusterInstance` in interface `Clusterer` **Overrides:**: `clusterInstance` in class `AbstractClusterer`
    :   **Parameters:**: `instance` - The instance to be assigned to a cluster **Returns:**: int The number of the assigned cluster as an integer **Throws:**: `java.lang.Exception` - If instance could not be clustered successfully

---


### toString

```
public java.lang.String toString()
```

:   return a string describing this clusterer

    :   **Overrides:**: `toString` in class `java.lang.Object`
    :   **Returns:**: a description of the clusterer as a string

---


### globalInfo

```
public java.lang.String globalInfo()
```

:   Returns a string describing this DataMining-Algorithm

    :   **Returns:**: String Information for the gui-explorer

---


### getTechnicalInformation

```
public TechnicalInformation getTechnicalInformation()
```

:   Returns an instance of a TechnicalInformation object, containing
    detailed information about the technical background of this class,
    e.g., paper reference or book this class is based on.

    :   **Specified by:**: `getTechnicalInformation` in interface `TechnicalInformationHandler`
    :   **Returns:**: the technical information about this class

---


### getRevision

```
public java.lang.String getRevision()
```

:   Returns the revision string.

    :   **Specified by:**: `getRevision` in interface `RevisionHandler` **Overrides:**: `getRevision` in class `AbstractClusterer`
    :   **Returns:**: the revision

---


### main

```
public static void main(java.lang.String[] argv)
```

:   Main method for testing this class.

    :   **Parameters:**: `argv` - should contain the following arguments: -t training file [-R repulsion]


---


|  |  |  |
| --- | --- | --- |
| **Overview** \| **Package** \| **Class** \| **Tree** \| **Deprecated** \| **Index** \| **Help** \| **Weka's home** | |  |
| **PREV CLASS**   **NEXT CLASS** | **FRAMES**    **NO FRAMES**     **All Classes** |  |
| SUMMARY: NESTED \| FIELD \| CONSTR \| METHOD | DETAIL: FIELD \| CONSTR \| METHOD |  |


---
